# Comparison of multi-organ CT image segmentation tools for whole-body [^18^F]FDG-PET/CT clinical imaging

**DOI:** 10.1007/s00259-025-07666-5

**Published:** 2025-11-27

**Authors:** Cameron Wheeler, Phyo H. Khaing, Eleonora D’Arnese, Adriana A.S. Tavares

**Affiliations:** 1 School of Informatics, University of Edinburgh, 10 Crichton St, Edinburgh, EH8 9AB Scotland, UK; 2 Centre for Cardiovascular Science, University of Edinburgh, 47 Little France Cres, Edinburgh, EH16 4TJ Scotland, UK; 3 Edinburgh Imaging, University of Edinburgh, 47 Little France Cres, Edinburgh, EH16 4TJ Scotland, UK

**Keywords:** Machine learning, Multi-organ segmentation, PET/CT imaging

## Abstract

**Abstract:**

Automated multi-organ segmentation looks to assist clinicians and researchers working with Positron Emission Tomography/Computed Tomography (PET/CT) imaging in streamlining the time-consuming, operator-dependent task of manual delineation. This study aimed to compare two state-of-the-art automated multi-organ CT segmentation tools with typically perceived “gold-standard” manual delineation.

**Methods:**

We compare Multiple-Organ Objective Segmentation (MOOSE) and TotalSegmentator against manual labels of six tissues on a dataset of 24 patients of lung cancer. We evaluated CT segmentation performance using the Dice–Sørensen Coefficient (DSC), Hausdorff Distance (HD), Average Symmetric Surface Distance (ASSD) and pixel-based metrics Precision and Recall. Alongside technical analysis, we perform evaluation using clinically relevant metrics including organ volume, mean standardised uptake value (SUV_mean_), maximum standardised uptake value (SUV_max_), and Hounsfield units.

**Results:**

Both MOOSE and TotalSegmentator produce overall comparable DSC results. Conversely, MOOSE and TotalSegmentator segmentation results in significantly different volumes and SUVs compared with manual delineation for the lungs, brain, and kidneys.

**Conclusion:**

Data presented here highlights the need to assess multi-organ segmentation tools performance using multi-pronged metrics beyond Dice-Sørensen scores.

**Supplementary Information:**

The online version contains supplementary material available at 10.1007/s00259-025-07666-5.

## Introduction

Typically, the delineation of volumes of interest (VOIs) in clinical PET/CT studies is performed manually by the interpreting clinician or radiographer [1]. This process is labour-intensive [2] and prone to both interand intra-operator variability, leading to inconsistent definitions of the same anatomical structure [1]. As quantitative, image-driven decision-making becomes clinical routine, the demand for accurate VOI delineation continues to grow, further increasing the workload on clinicians. Conventional clinical PET/CT workflows often record only a handful of statistics, thereby leaving much of the rich information captured within the images untapped [3]. Furthermore, with the introduction of total-body PET/CT (TB-PET/CT) systems into both the clinical and research environments, the ability to capture the entire axial extent of the patient in a single bed position now enables whole-system analyses in high resolution [4]. This is particularly advantageous for multi-organ conditions such as metastatic cancer where inter-organ relationships have not been previously studied en masse. To fully exploit the capabilities TB-PET/CT systems offer in both research and clinical settings, organ-level delineations must be produced rapidly and reproducibly, requirements that manual contouring simply cannot meet. As such, the application of automated multi-organ segmentation aims to address the issues in the VOI delineation process, increasing image throughput and reducing the current manual stochasticity involved. Moreover, automation in the segmentation process has the potential to galvanise downstream analyses, including network analysis and kinetic modelling [5, 6].

Among the various automated multi-organ segmentation approaches, two methodologies Multiple-Organ Objective Segmentation (MOOSE) [7] and TotalSegmentator (TS) [8] have emerged as state-of-the-art (SOTA). Both methods leverage the nnU-Net framework [9] as their underlying architecture. However, they differ in training methodology and dataset, which leads to differences in model configurations and weights. Recently, Julie et al. [10] compared MOOSE and TotalSegmentator using a dataset of metastatic breast cancer. However, gold-standard labels were not generated for this study and as a result, direct comparison against a verified gold-standard is not available, instead the authors focus solely on the degree of agreement between the two methods and differences in clinical feature values.

Concurrent with this work, we evaluate MOOSE and TS in a cohort of clinical stage IIB/III non-small cell lung carcinoma (NSCLC) patients [11]. Each automated segmentation is compared with expert manual delineations using established technical metrics—Dice–Sørensen Coefficient (DSC), Hausdorff Distance (HD), Average Symmetric Surface Distance (ASSD), as well as voxel-wise Precision and Recall. In addition, we quantify potential downstream bias by comparing PET/CT-derived metrics (e.g., segmented volume, Hounsfield units, and standardised uptake values) between automated and manual VOIs.

## Methods

To compare MOOSE (v2.6.3) [7], TotalSegmentator (v2.2.1) [8] and manual segmentation methods, we select 24 patients (sex 17 M/7F, age 59 ± 9.5, weight (kg) 74.6 ± 15.4, height (cm) 170 ± 8.5) with clinical stage IIB/III NSCLC from a multi-center clinical trial dataset ACRIN 6668 available on the TCIA [11]. Of the 250 patients enrolled in the study, the 24 patients selected for this study are a subset of the patients included in Ronghe et al. [12] and as such adhere to their inclusion criteria. Images were selected based on the presence of wholebody PET/CT images and corresponding gold-standard labelling, alongside having no evidence of metastatic disease. All images included must have been fully compatible with MOOSE and TotalSegmentator’s imaging pipelines. All patients were imaged using both CT and [^18^F]FDG-PET prior to receiving treatment. CT imaging was acquired using helical and/or multi-slice technology with a slice thickness of ≤ 0.5 cm, ensuring coverage of the thorax and gross tumour volume. It should be noted that, as the images were taken as part of a clinical routine, not all images in the dataset encompass the full body field of view; in some cases, the head or lower legs are truncated. PET imaging was performed following 4 h fasting with a blood glucose level of ≤ 200 mg/dl. An [^18^F]FDG dose of 5.18–7.77 MBq/kg was given with emission imaging starting 50–70 min post-injection.

Reconstruction of images within the study was dependent on the scanner utilised. A reconstruction pipeline of OSEM reconstruction with 8 subsets and 2 iterations, followed by smoothing with a 6-mm 3D Gaussian kernel was recommended by the trial organisers. For details regarding specific features of the dataset utilised within this study (scanner manufacturer, model and imaging features), please reference Supplementary Table S1. With a focus on image pre-processing, both MOOSE and TotalSegmentator are built upon the nnU-Net framework [9], which includes automated pre-processing steps including resampling, normalisation, and cropping as part of the inference pipeline. No additional manual pre-processing was applied to the PET/CT images prior to segmentation, in order to preserve the clinical authenticity of the data and to evaluate the models’ performance under representative real-world conditions. Furthermore, predicted masks are automatically resampled into the original image space following inference.

While both MOOSE and TS can generate segmentation masks for over 100 target structures, they do not segment tumours as distinct tissues. Our objective, therefore, is organ segmentation in which any tumour present is excluded from the corresponding organ mask. Our gold-standard included six tissues (brain, left/right lung, left/right kidney, and liver) generated by a clinician with seven years of experience, using PMOD 3.7 (PMOD Technologies, Switzerland). The target structures were selected based on data availability from clinician segmentation and segmentation difficulty. These structures are also amply investigated [2, 13, 14], therefore, enabling a comparison with previously published data. The clinician performed manual delineations using both PET and CT imaging modalities. During this process, pulmonary tumours were intentionally excluded from the final segmentation masks. No tumours were present in the other anatomical regions. Analysis of images found the presence of disease within the left and right lung is split 9/15 respectively.

Within this study, the automated segmentation methodologies generate their predictions using the CT domain only. DICOM segmentation masks generated by both MOOSE and TS were imported into PMOD 4.2, and VOI delineations were generated for comparison and metric computation. We defined SUV_*mean*_ as mean within the VOI and SUV_*max*_ as the mean of the top five hottest voxels within a VOI in order to provide a stable measurement of SUV uptake. Both are normalised to the injected dose and patient weight. Dice-Sørensen Coefficient (DSC), Precision (Pr), Recall (Rc), Hausdorff Distance (HD), and Average Symmetric Surface Distance (ASSD) were computed using Python 3.10.15 and MONAI [15]. To ensure optimal performance, no limitations were imposed on the automated methods such as the use of the “–fast” option in TotalSegmentator. All inference was conducted on an NVIDIA A100 GPU with 40 GB of memory.

We set a significance threshold of *p* ≤ 0.05 for all statistical analyses. Normality was assessed using the Shapiro-Wilk test. For non-normal data, we used the Wilcoxon test for two-group comparisons and the Friedman test with Dunn’s multiple comparisons correction for three-group comparisons. For normal data, we applied a Repeated-Measures One-Way ANOVA with Tukey’s correction. Analyses with missing values in cerebral segmentation were conducted using a Mixed-Effects model with Tukey’s correction. Outliers were identified using the ROUT method (*Q* = 0.1%). All statistical tests and graphs were performed using GraphPad Prism version 10 (GraphPad Software, United States of America).

## Results

### MOOSE and totalsegmentator produced VOIs with comparable DSC

**Fig. 1:**
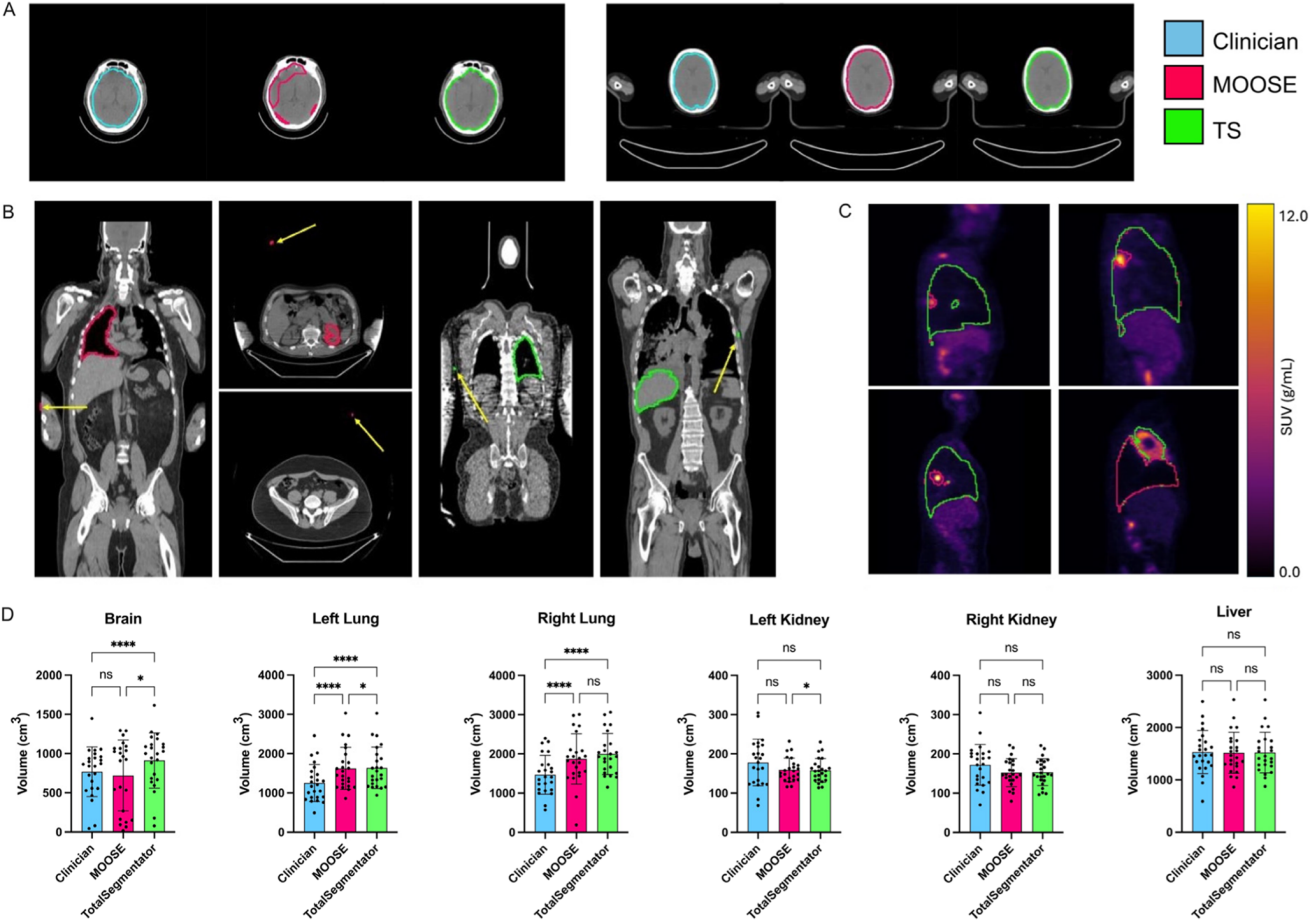
[^18^F]FDG-PET and CT delineation examples with volumetric comparisons between clinician, MOOSE and totalsegmentator generated VOIs:** A**: Examples where MOOSE struggled/did not struggle to capture the full brain VOI. **B**: Examples of false positive predictions far from the target VOI. Arrows point to false positive prediction. **C**: MOOSE was able to capture tumors present within the right lung, avoiding inclusion in the VOI. **D**: Volumetric comparisons between delineation methodologies. Significance Testing: One Way ANOVA. **** indicates *p <* 0.0001, * indicates *p <* 0.05. MOOSE vs. TS Brain: *p* = 0.013, MOOSE vs. TS Left Lung: *p* = 0.043, MOOSE vs. TS Left Kidney: *p* = 0.049

Both TS and MOOSE perform respectfully with mean dataset DSC of 0.79 ± 0.05 and 0.76 ± 0.07, respectively with no significant differences indicating that MOOSE and TS are fairly robust to the variance of patient position, disease progression, and scanner vendor within our dataset. Brain segmentation DSC was the most variable metric between methods. Analysis showed that DSC is significantly different between MOOSE and TS in the segmentation of the brain (Wilcoxon *p <* 0.0001) (Table 1). Post-hoc analysis revealed cases where MOOSE underestimated or missed delineation of the brain (Fig. 1A). For all other organs, we found no significant difference in DSC.

**Table 1:** Mean Dice-Sørensen coefficient, Hausdorff distance, average symmetric surface distance, precision, and recall between MOOSE and totalsegmentator computed using gold-standard annotations on the CT modality. **Bold-face **indicates the best performance. ↑ higher is better. ↓ lower is better

Organ	Dice Score ↑		HD (mm) ↓		ASSD (mm) ↓		Precision ↑		Recall ↑	
	TS	MOOSE	TS	MOOSE	TS	MOOSE	TS	MOOSE	TS	MOOSE
Brain	**0.87 ± 0.11**	0.67 ± 0.32	**28.07 ± 46.95**	45.33 ± 51.08	**2.29 ± 2.46**	6.53 ± 7.76	**0.79 ± 0.12**	0.74 ± 0.18	**0.97 ± 0.10**	0.71 ± 0.34
Left Lung	0.84 ± 0.58	**0.85 ± 0.05**	87.46 ± 174.80	**34.80 ± 40.35**	3.21 ± 0.51	**3.06 ± 0.49**	0.74 ± 0.08	0.74 ± 0.07	**0.99 ± 0.02**	0.98 ± 0.02
Right Lung	0.83 ± 0.093	0.83 ± 0.13	**34.48 ± 12.77**	65.22 ± 113.16	4.07 ± 1.58	**3.47 ± 1.43**	0.72 ± 0.12	0.75 ± 0.07	**0.99 ± 0.01**	0.95 ± 0.17
Left Kidney	0.70 ± 0.07	0.70 ± 0.07	**24.85 ± 9.34**	162.1 ± 340.7	**3.35 ± 1.27**	3.57 ± 1.39	**0.77 ± 0.17**	0.76 ± 0.17	0.69 ± 0.10	0.69 ± 0.10
Right Kidney	**0.70 ± 0.08**	0.69 ± 0.09	**27.00 ± 9.49**	96.22 ± 191.89	**3.45 ± 1.39**	3.60 ± 1.67	**0.75 ± 0.14**	0.75 ± 0.15	**0.68 ± 0.12**	0.67 ± 0.13
Liver	0.85 ± 0.07	0.85 ± 0.07	69.45 ± 54.97	**60.59 ± 37.19**	**3.25 ± 1.59**	3.29 ± 1.62	0.85 ± 0.08	0.85 ± 0.08	0.85 ± 0.09	0.85 ± 0.09

In addition to overall similarity, consistent findings emerged from the pixel-based analysis (Table 1). TS outperformed MOOSE in cerebral segmentation for both precision and recall, with percentage point difference of 5% and 26%, respectively. Within the non-cerebral tissues, we find much smaller differences between the two methods. In the right lung, MOOSE achieved higher precision (3%), whereas TS demonstrated superior recall (4%). For the left lung, TS exhibited a modest advantage in recall (1%), while both methods achieved identical precision scores. In the kidneys, TS showed a slight improvement in left kidney precision (1%) and higher recall in the right kidney (1%). For all other tissues, we found no difference in pixel based analysis indicating the same technical performance.

### Tumors and image artifacts drive segmentation errors

TS brain segmentation demonstrates substantially lower maximum error rates than MOOSE. Figure 1A shows a case where MOOSE failed to capture the full brain within its mask. Our dataset contains patients where truncation of the brain is seen. However, further analysis found errors were not a result of truncation; with MOOSE performing well in many cases (Fig. 1A), whether heavy truncation was present or not. Similarly, kidney segmentation is a noticeable difference in MOOSE compared to TS. Further analysis revealed this arises from MOOSE inadvertently segmenting artifacts in certain images (Fig. 1B), rather than a foundational inability to accurately segment the kidneys. Comparing the lungs, MOOSE and TS were both making false positive predictions (Table 1; Fig. 1B). Liver segmentation errors appeared to be similar across the methods. We observed a closer similarity in ASSD results between MOOSE and TS (Table 1), suggesting average delineation errors between the methods are similar across the non-cerebral organs when compared to maximal errors.

### MOOSE and totalsegmentator show organ-dependent volumetric bias compared to manual delineation

**Fig. 2:**
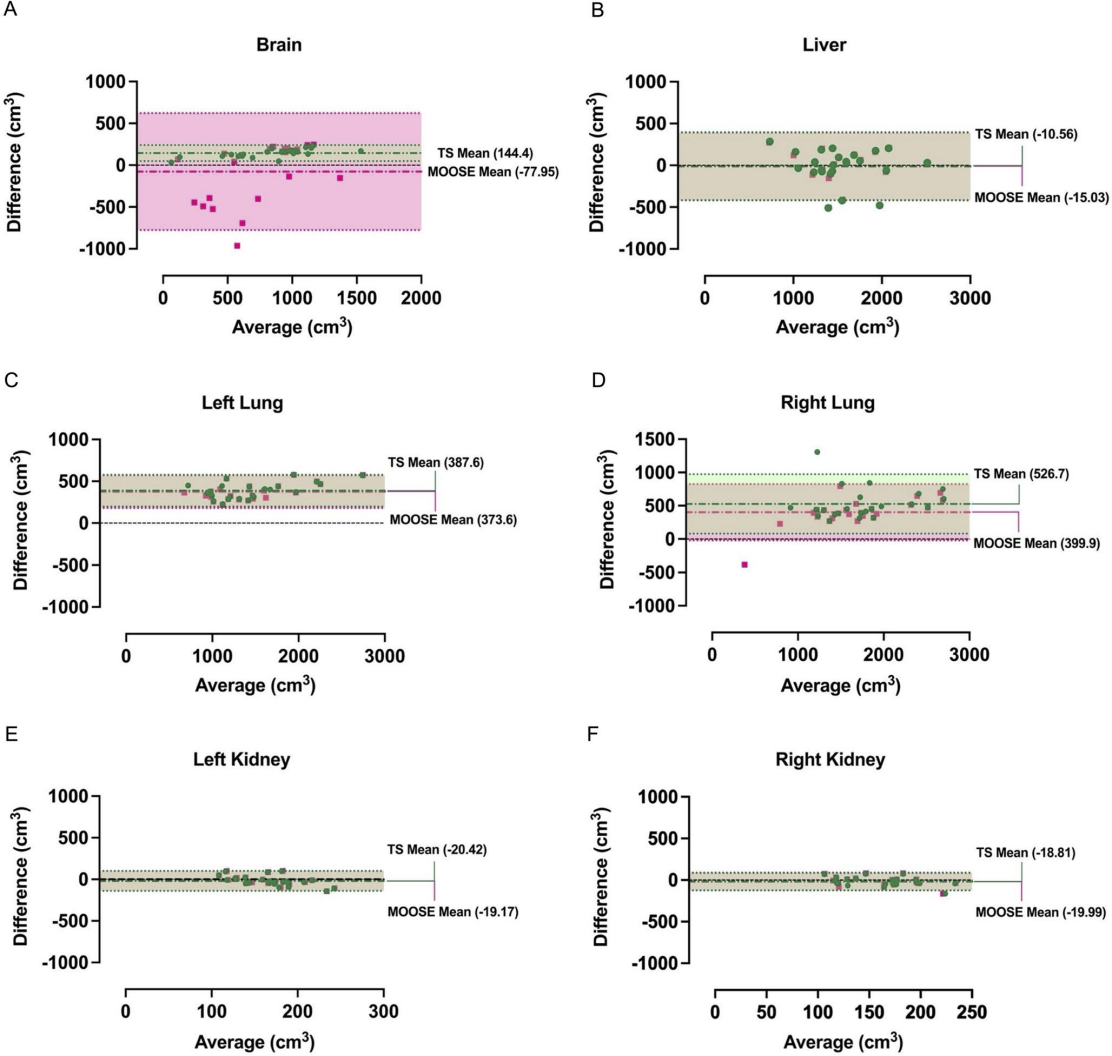
Analysis of volumetric estimate bias of clinician vs. MOOSE and clinician vs. totalsegmentator: **A-F**: Bland-altman plots for each organ within our dataset. Mean values indicate the mean difference in volumetric estimate between our gold-standard and the estimated volume from MOOSE or totalsegmentator. Colours: Red - MOOSE, Green - TotalSegmentator

With the exception of the left kidney MOOSE vs. TS comparison, liver and kidney volumes delineated by both MOOSE and TS showed no significant differences between each other or the gold-standard annotation (Fig. 1D). We found significant differences between our gold-standard and both methods in lung volume. Interestingly, significant differences between MOOSE and TS are only present in the left lung, despite the small mean difference of 15cm^3^. For brain volumes, we report a significant difference between TS and our gold-standard alongside a significant difference between MOOSE and TS; a significant difference was not found between MOOSE and gold-standard estimates. The Bland-Altman plots (Fig. 2) confirmed the greatest bias in volume estimates by both methods was in the lungs. Conversely, the least biased volume estimates by both TS and MOOSE was observed in the kidneys. Data showed that MOOSE brain segmentation displays greater differences versus gold-standard manual annotation with a larger absolute difference of limit intervals (1399.2*cm*^3^) in comparison with TS (191.57cm^3^) indicating a high variability in differences between our gold-standard and MOOSE brain volumes.

### SUV and HU outcomes are biased in an organ-dependent manner versus manual delineation

**Fig. 3:**
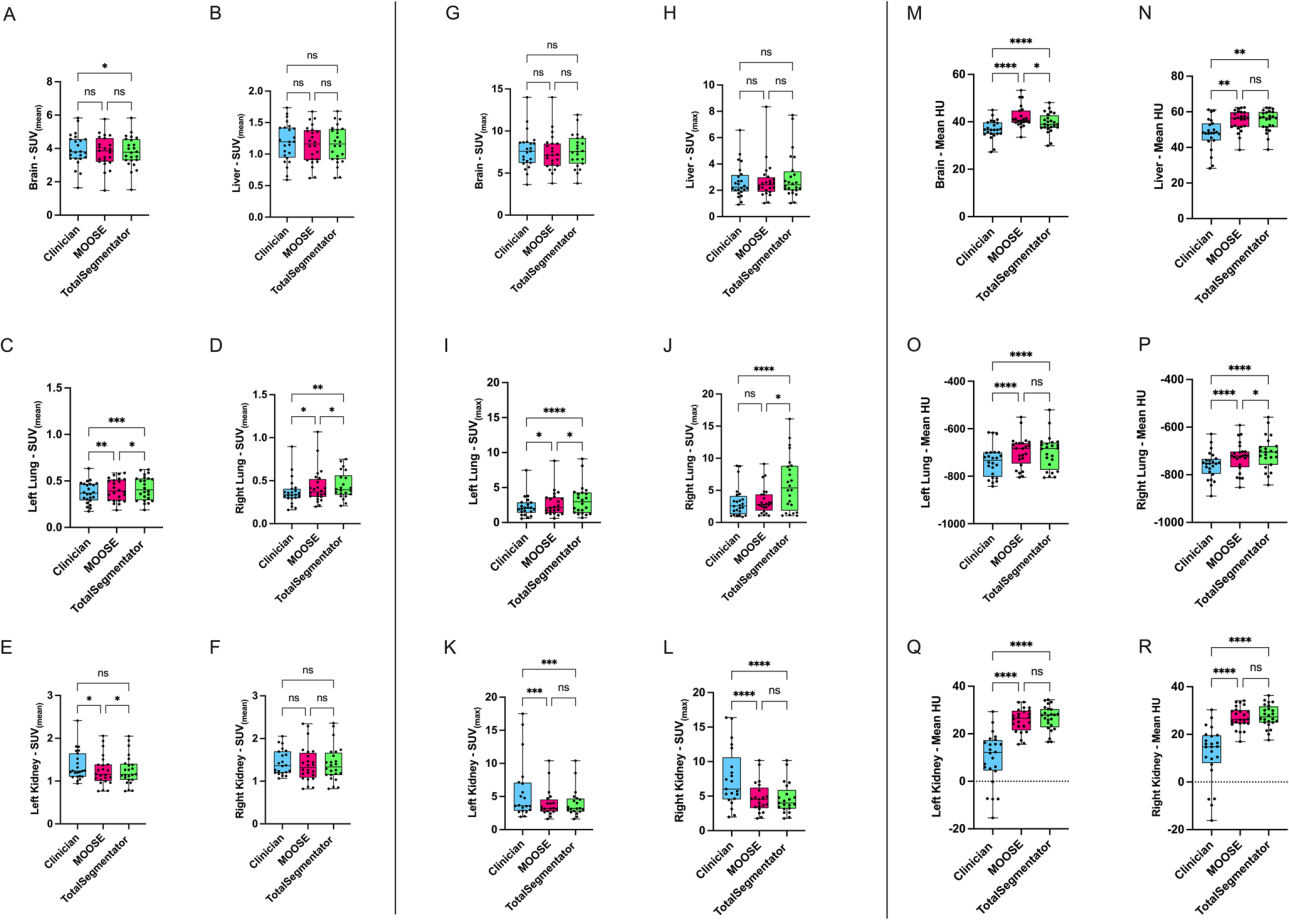
[^18^F]FDG-PET SUV_*mean*_, SUV_*max*_ and HU of Clinician, MOOSE and TS Volumes of Interest:** A-F**: SUV_*mean*_ distributions for each target. **G-L**: SUV_*max*_ distributions for each target. **M-R** HU distributions for each target. Significance Testing: SUV_*mean*_, HU One Way Anova, SUV_*max*_ Friedman. **** indicates *p <* 0.0001, *** indicates *p <* 0.001, ** indicates *p <* 0.01, * indicates *p <* 0.05. **A**: Clinician vs. TS *p* = 0.048, **C**: MOOSE vs. TS *p* = 0.02 **D**: Clinician vs. MOOSE *p* = 0.0106 MOOSE vs. TS *p* = 0.048, **E**: Clinician vs. MOOSE *p* = 0.039 MOOSE vs. TS *p* = 0.016, **I**: Clinician vs. MOOSE *p* = 0.047, MOOSE vs. TS *p* = 0.012, **J**: MOOSE vs. TS *p* = 0.011, **M**: MOOSE vs. TS *p* = 0.012, **P**: MOOSE vs. TS *p* = 0.032

Investigating the impact of segmentation on clinical outcomes showed that SUV_*mean*_ results were significantly different in the lungs (Fig. 3C & D). Some differences were observed between brain manual segmentation and TS (Fig. 3A) and MOOSE versus TS and gold-standard vs. MOOSE segmentation of the left kidney (Fig. 3E). Liver and right kidney SUV_*mean*_ outcomes were not significantly affected by the segmentation method used. For SUV_*max*_, significant differences were present within the lungs (Fig. 3I & J), bar goldstandard vs. MOOSE in the right lung. Of note, TS mean SUV_*max*_ in the right lung was considerably higher than the gold-standard and MOOSE (Fig. 3J). In contrast to SUV_*mean*_ we found significant differences between our gold-standard and both automated methodologies in the kidneys (Fig. 3K & L). We observe no significant difference in SUV_*max*_ for brain or liver VOIs. HU analysis shows significant differences between our gold-standard annotations and the automated methods across all organs within our dataset (Fig. 3MR). This is particularly drastic in the kidneys where our gold-standard displays a lower mean of HU values when compared to MOOSE and TS (Fig. 3Q, R). No significant differences between MOOSE and TS were found, except for the brain (Fig. 3M) and right lung (Fig. 3P).

## Discussion

This study compared two automatic multi-organ segmentation methodologies, MOOSE [7] and TotalSegmentator [8], using a dataset of clinical stage IIB/III NSCLC patients. Both methodologies showed comparable performance in the thorax and abdomen. We found MOOSE underestimated the volume of all organs except the lungs similar to results found in [10]. However, the degree of underestimation for the liver was relatively minor. Unlike Julie et al. [10] MOOSE PET SUV_*max*_ is only significantly lower than TS in the lungs. MOOSE showed strength in capturing disease presentation within the lungs, likely the cause of the lower SUV_*max*_. Segmentation within the lungs is already perceived as a difficult task due to breathing artifacts [16], made further difficult with the presence of disease that can alter the anatomical structure [17]. With updates to its training dataset from release (50 to 1.5k images in MOOSE 2.0) the heterogeneity in diseased presentation seen during MOOSE’s training could have influenced this difference [17]. Comparing this to TS’s 1082 training images [8] of which only ∼200 images contain tumours as the main pathology [8] we observed that TS’s lung delineation often followed a standardised pattern associated with normal lung anatomy. Achieving comprehensive coverage of clinical heterogeneity in training is a herculean task [7] of which training data is a vital aspect. Discussion on this issue is currently centered on generalising between different disease types (cancer) alongside the need to generalise across distinct diseases [18]. This underscores the critical need for research into pipelines designed with disease-agnostic segmentation capabilities in mind.

Within our dataset, several cases exhibited –to differing extents– truncation of the brain. We acknowledge that any study involving cerebral structures necessitates complete cranial coverage, which was not uniformly present in our cohort. However, our objective was to evaluate both MOOSE and TotalSegmentator on images that reflect the variability commonly observed in routine clinical practice, including substantial variation in imaging field-of-view. Although we observed occasional underperformance of MOOSE in specific cases, there was no consistent evidence to attribute this solely to cranial truncation. In fact, in the majority of truncated cases, MOOSE behaved as expected. Notably, at the time of our analysis, Sundar et al. [7] had only recently released updated model weights that extended brain segmentation capabilities to total-body CT images, whereas prior versions of MOOSE supported brain segmentation exclusively in the PET domain, where the brain constitutes a major part of the field of view. We interpret some of the observed inconsistencies as indicative of early-stage limitations in the updated model, which we anticipate will be addressed in future iterations of the tool.

While the DSC scores indicated minimal differences in non-cerebral organs, analysis using clinical metrics, revealed nuanced distinctions between MOOSE and TotalSegmentator. Specifically, PET-based evaluations suggest that MOOSE demonstrates a greater ability to detect and delineate tumours within the lungs. These differences are not captured when comparing the two methods using overlap and similarity-based metrics, but is observable when utilising PET SUV_*mean*_ and PET SUV_*max*_. In their article Sundar and Beyer [18] discuss an example where improvements in DSC over 0.70, could yield limited improvements in prognostic accuracy. We show that despite achieving a DSC over 0.70, clinically applicable metrics such as SUV_*mean*_ and SUV_*max*_ used in prognosis tasks [19, 20] are significantly different from our gold-standard and, as a result, prognosis results could vary. This indicates that a hyper-focus on optimizing DSC alone is insufficient when developing tools for the clinic or research. Furthermore, it aligns with Sundar and Beyer [18], who suggest that future research should aim to identify a threshold value at which clinical endpoints show negligible differences between automated and manual approaches, regardless of DSC improvements. Currently, many papers within the field of medical segmentation assess their proposal using similarity-based metrics such as DSC or Intersection over Union (IoU) and occasionally evaluate further with error-based metrics such as HD, ASSD or normalised surface distance [21–23]. Maier-Hein et al. [24] suggests using an ’overlap and boundarybased approach’ within semantic segmentation evaluation. However, we further suggest that authors evaluate performance using more clinically applicable metrics alongside their typical suite of similarity/error-based evaluation scores when developing methodologies to capture a more realistic picture of the tools being produced. This suggestion is consistent with the recommendations of Jha et al. [25], who also argue that technical metrics, referred to as “figures of merit” should be complemented by evaluations based on clinical task performance in order to comprehensively assess machine learning applications in medical imaging.

Our analysis demonstrates that MOOSE and TotalSegmentator perform similarly when assessed using technical metrics. However, we identify distinct differences in their behaviour when evaluated from a clinical perspective. For example, our work shows differences in volumetric estimates of the target structures alongside nuanced differences, particularly around the ability to handle pathological presence within the diseased tissue as shown in the SUV_*mean*_, SUV_*max*_ and HU endpoints. Compared to the performance reported in their respective original publications, both models show a reduction in performance in our study. While we attribute this primarily to the inclusion of patients with pathological findings unlike the largely healthy cohorts used in the original studies, it would be incorrect to consider this the sole factor. We observe performance drops across all organs, regardless of pathology. This underscores a fundamental challenge in multi-organ segmentation for medical imaging: the inherent variability in clinical annotations [26]. Even among experienced clinicians, differences in interpretation and delineation are common, introducing a level of uncertainty that is difficult to eliminate entirely [26]. Both models were developed using an iterative learning strategy [27], in which the model is iteratively refined through cycles of training, correction, and retraining on their respective datasets. While their strategy differs, MOOSE detects errors through the use of out-of-distribution analysis, whereas TotalSegmentator refines all masks predicted in that round. If the corrections are consistently performed by the same set of clinicians, this inherently biases the models toward their specific interpretation of the segmentation task. When such a model is evaluated against segmentations produced by other clinicians, who may have different delineation practices, discrepancies can arise, not necessarily due to model failure, but due to inter-observer variability in what constitutes the “correct” mask.

A limitation of our study is the use of a single clinician for the generation of gold-standard VOIs. Interclinician variability remains a significant challenge in medical image segmentation, consequently, the results presented in this study could vary if the same methodology were applied with a different clinician. This is highlighted in the kidney, where our gold-standard often includes the renal pelvis, likely a result of using purely PET for VOI generation, while it is not the case for MOOSE and TotalSegmentator. Furthermore, the use of a single annotator limits our ability to assess inter-observer variability and to determine whether the differences observed in our study may be attributable to such variability. Regarding the liver, Cai et al. [28] reported approximately 3% volumetric difference between ground truth and three commercial packages in non-diseased livers. For kidneys, Joskowicz et al. [29] quantified inter-observer variability across multiple structures and found a mean 8.8% difference in kidney volumes between two randomly selected observers (from 11 annotators). With respect to the lungs, the primary pathological organ in our cohort, we did not identify NSCLC-specific inter-observer studies. In healthy or non-oncologic settings, Kaza et al. [30] observed variability *<* 6% across three observers, and Nemec et al. [31] reported inter-observer variability for healthy lung volumes ranging from 0.20 to 6.45%. Algorithm–human comparisons in disease models show similarly small discrepancies, for example Maiello et al. [32] found an approximately 5% difference relative to a two-observer consensus in a pig COVID-19 model. While these cohorts and comparators are not a like-for-like comparison with our NSCLC population, collectively they indicate that typical inter-observer or inter-software variability for organ volume is generally on the order of only a few percent points. In our cohort, the mean absolute relative differences in organ volumes between each algorithm and the clinician ground truth were substantially larger for the lungs and kidneys than for the liver. For the kidneys, mean differences were 31.9% (MOOSE) and 31.7% (TS) on the left and 28.2% and 27.2% on the right. For the lungs, the mean differences were 32.9%/34.6% (left) and 31.8%/43.3% (right). By contrast, the liver showed smaller mean differences of 10.6% (MOOSE) and 10.5% (TS). Our results do indicate that the differences are outside of inter-observer variability found within the literature. While we acknowledge that these are not strictly like-for-like comparisons, we do believe that the relative differences are large enough to conclude that the differences in results are not exclusively due to inter-observer variability between our gold-standard and the automated methodologies. Using multiple clinicians to generate gold-standards alongside algorithms such as STAPLE [33] could be used to generate a consensus [7] and enable the further study of inter-operator variability and a deeper analysis into the subject. A final limitation within our study is the number of images used for evaluation. Our dataset is comparatively small when compared to [10]. However, the cost of medical label generation is an established issue within automated segmentation, as such the dichotomy of dataset size and access to labels cannot be avoided.

## Conclusion

To conclude, our findings underscore the substantial progress being made by both MOOSE and TotalSegmentator in multi-organ segmentation. On a dataset of 24 NSCLC patients, we demonstrate that from a technical perspective, both methods perform comparably in non-cerebral organs, including diseased lung tissue. At the same time, we highlight areas where further refinement is needed to improve their applicability in both clinical and research settings. Furthermore, this study emphasises the importance of employing clinically relevant, task-specific metrics when evaluating automated segmentation pipelines, as these offer a more comprehensive and nuanced understanding of model behaviour.

## Supplementary Information

Below is the link to the electronic supplementary material.

ESM 1 (PDF 1.23 MB)

## Data Availability

The data utilised within the study is available on The Cancer Imaging Archive, under the project name ACRINNSCLC-FDG-PET | ACRIN 6668 https://www.cancerimagingarchive.net/collection/acrin-nsclc-fdg-pet/.
