# Supplementary material for: Comparison of multi-organ CT image segmentation tools for whole-body [18F]FDG-PET/CT clinical imaging
Source: Eur J Nucl Med Mol Imaging. 2025 Nov 27;53(5):3172–81. doi: 10.1007/s00259-025-07666-5 (PMC13013167; doi:10.1007/s00259-025-07666-5)
Supplement: Supplementary file 1 — (PDF 1.23 MB) [file 259_2025_7666_MOESM1_ESM.pdf]

Supplementary Material

Table S1: Scanner manufacturer, model, and voxel characteristics across all 24 patients included in the study. Counts represent the number of patients associated with each characteristic. Axial slice size refers to the in-plane matrix dimensions.

	Modality			
	CT	Value	PET	Value
Scanner Manufacturer	SIEMENS (S)	10 14	CPS	10 14
	GE		GE	
	**Total**	24	**Total**	24
Scanner Model	Discovery ST (GE)	3	Discovery ST (GE)	3
	Discovery LS (GE)	6	Discovery LS (GE)	6
	Discovery RX (GE)	1	Discovery RX (GE)	1
	Discovery STE (GE)	4	Discovery STE (GE)	4
	Emotion 6 (S)	2	1023 (CPS)	4
	Sensation 16 (S)	2	1024 (CPS)	2
	Emotion Duo (S)	6	1080 (CPS)	4
	**Total**	24	**Total**	24
# Of Slices	$100 < x \leq 200$	1	$100 < x \leq 200$ $200 < x \leq 250$	2 6
	$200 < x \leq 250$	5		
	$250 < x$	18	$250 < x$	16
	**Mean [Min-Max]**	299 [171-584]	**Mean [Min-Max]**	272 [171-390]
Pixel Spacing	$(0.1 \times 0.1) < x \leq (0.5 \times 0.5)$	0	$(2.0 \times 2.0) < x \leq (3.0 \times 3.0)$	3
	$(0.5 \times 0.5) < x \leq (1 \times 1)$	23	$(3.0 \times 3.0) < x \leq (4.0 \times 4.0)$	6
	$(1 \times 1) < x$	1	$(4.0 \times 4.0) < x$	15
	**Mean [Min-Max]**	0.992 [0.976-1.37]	**Mean [Min-Max]**	4.43 [2.63-5.47]
Z-Spacing	$1.0 < x \leq 1.5$	1	$1.0 < x \leq 1.5$	0
	$1.5 < x \leq 2.0$	0	$1.5 < x \leq 2.0$	0
	$2.0 < x \leq 2.5$	5	$2.0 < x \leq 2.5$	3
	$2.5 < x \leq 3.0$	0	$2.5 < x \leq 3.0$	0
	$3.0 < x \leq 3.5$	11	$3.0 < x \leq 3.5$	13
	$x > 3.5$	7	$x > 3.5$	8
	**Mean [Min-Max]**	3.27 [1.5-4.25]	**Mean [Min-Max]**	3.54 [2.5-5.0]
Slice Thickness	3.0	1	2.0	4
	3.75	8	3.27	8
	4.0	1	3.37	6
	5.0	14	4.25	6

	**Mean [Min-Max]**	4.46	**Mean [Min-Max]**	3.33 [2.0-4.25]
Axial Slice Size	512 x 512	24	128 x 128<br>256 x 256	21<br>3

Brain Frequency Distribution

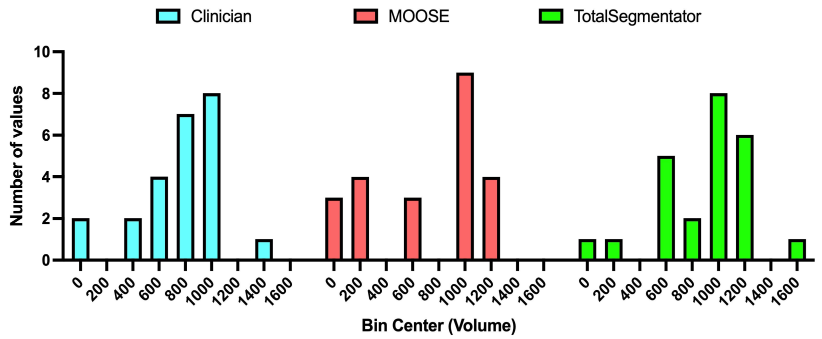

Liver Frequency distribution

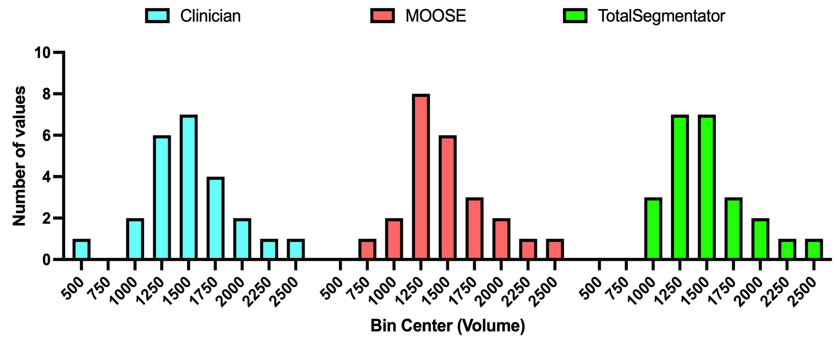

Left Lung Frequency distribution

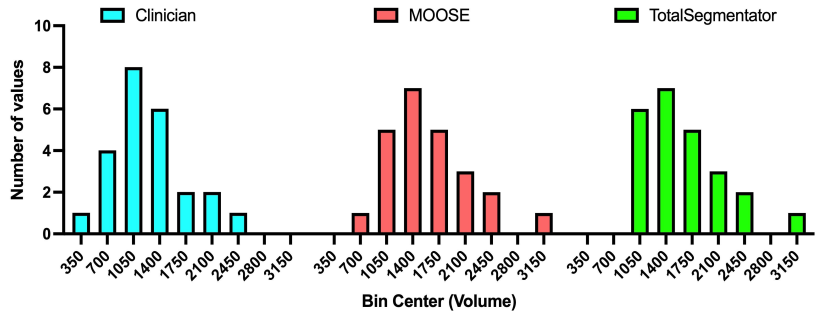

Right Lung Frequency distribution

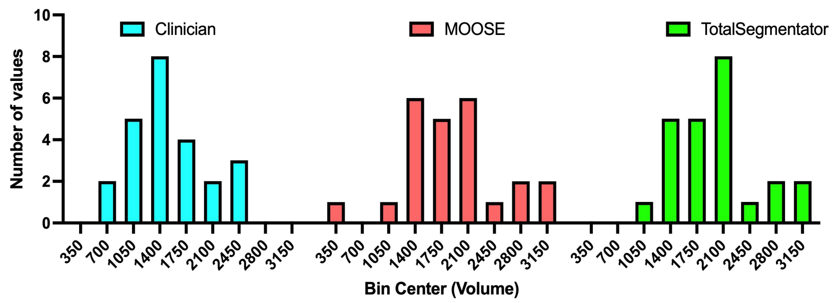

Fig. S1: **Volume Histograms of Target Structures:** From top to bottom: brain, liver, left lung, and right lung. While the non-cerebral organs exhibit normally distributed volumetric values, the brain demonstrates greater scatter, reflecting variability introduced by truncation effects within the dataset.

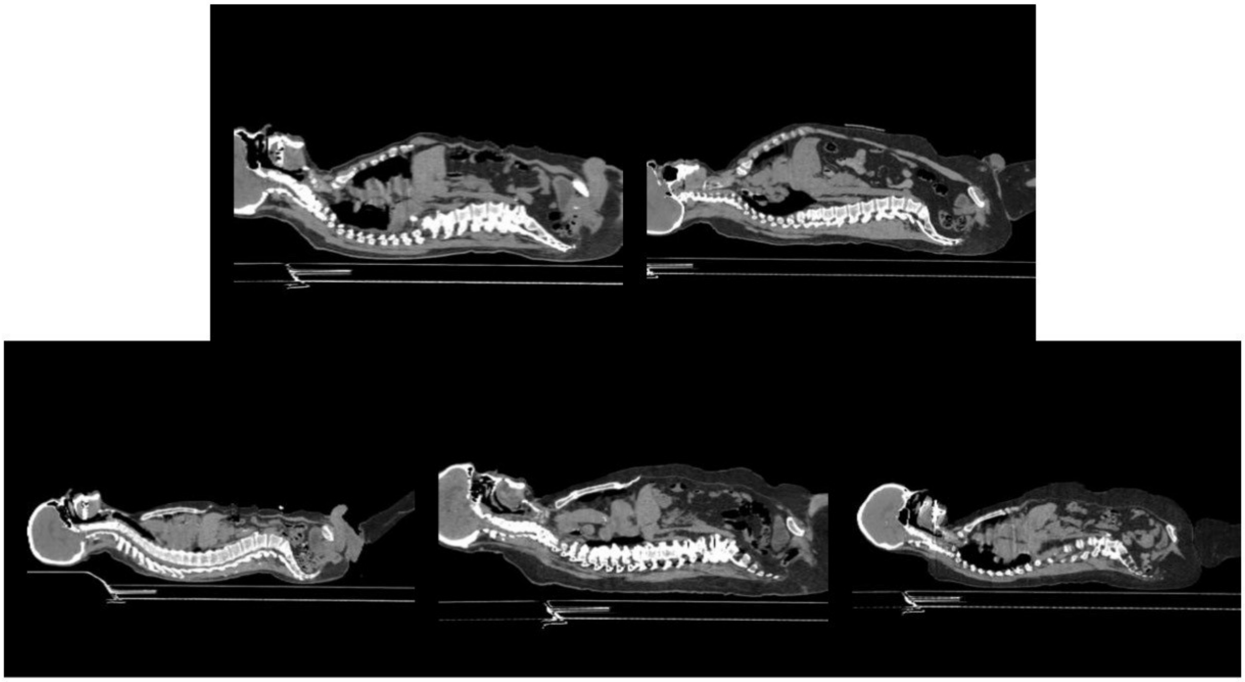

Fig. S2: **Sample Images:** Visualisation of five patients from the ACRIN 6668 dataset utilised within the study. Various levels of truncation of the brain can be found from heavy truncation to no truncation.
